# An Experimental Test of whether the Defensive Phenotype of an Aphid Facultative Symbiont Can Respond to Selection within a Host Lineage

**DOI:** 10.1371/journal.pone.0111601

**Published:** 2014-11-14

**Authors:** Ailsa H. C. McLean, H. Charles J. Godfray

**Affiliations:** Department of Zoology, University of Oxford, Oxford, United Kingdom; University of California, Berkeley, United States of America

## Abstract

An experiment was conducted to test whether parasitoid resistance within a single clonal line of pea aphid (*Acyrthosiphon pisum*) might increase after exposure to the parasitoid wasp *Aphidius ervi*. Any change in resistance was expected to occur through an increase in the density of protective symbiotic bacteria rather than genetic change within the aphid or the bacterial symbiont. Six aphid lineages were exposed to high parasitoid attack rates over nine generations, each line being propagated from individuals that had survived attack; a further six lineages were maintained without parasitoids as a control. At the end of the experiment the strength of resistance of aphids from treatment and control lines were compared. No differences in resistance were found.

## Introduction

Animals are not as well defended against parasites and diseases as is physiologically possible and additive genetic variation in resistance is frequently observed [1], [2]. The level of resistance exhibited is likely to reflect a number of different trade-offs between immune function and other aspects of the organism's life-history including development time [3], competitive ability [4] and reproductive output [5]. Artificially strengthening selection for resistance in an experimental setting can prove useful in understanding how this variation arises and what determines the patterns of resistance observed in the field [3], [4].

Parasitoids and their insect hosts provide excellent systems for studying the evolution of resistance because their development is intimately intertwined, with survival of one dependent upon the death of the other [6]. In aphids, a major component of the defensive response against endoparasitoids is provided by facultative endosymbiotic bacteria, in particular *Hamiltonella defensa* [7], [8]. The ability to confer resistance requires *Hamiltonella* to be infected by particular strains of a bacteriophage, termed APSE (*Acyrthosiphon pisum* Secondary Endosymbiont [9]), which encode a number of toxin genes whose products are thought to play a role in attacking the developing parasitoid [10], [11]. In other respects, aphids appear to have a reduced immune system relative to most insects [12], [13], but whether this is a cause or a consequence of their association with symbiotic bacteria is unknown. Nevertheless, aphids may possess considerable intrinsic as well as symbiont-conferred resistance to parasitoid wasps [14].

The braconid parasitoid wasp, *Aphidius ervi*, has been shown to evolve higher virulence (defined as successful parasitism) when populations were maintained on a line of aphids infected with a protective strain of *Hamiltonella* [15]. In a separate study, adults of *A. ervi* were found to adapt to parasitize aphids with symbiont-associated resistance by laying multiple eggs in a single individual (self-superparasitism) [16]. Only one adult parasitoid can emerge successfully from a single parasitized aphid, but it is thought that laying several eggs can dilute and hence overwhelm host defences. Given the potential for parasitoids to evolve rapidly in response to host defences, it would be of interest to know whether aphid defences can evolve to respond to greater parasitoid challenge.

Aphid populations could achieve better symbiont-associated resistance through a variety of routes. First, they might acquire more highly protective bacteria. The facultative symbiont *Hamiltonella* is transmitted maternally with almost perfect fidelity in the asexual phases of aphid reproduction, and is also inherited during the sexual generation [17]. It can also be transmitted horizontally between individuals of the same [18]–[20] or different [21] species. Exactly how horizontal transfer occurs is not currently understood (there is experimental evidence that parasitoids are capable of transmitting *Hamiltonella* via oviposition [22]) but acquisition of novel symbionts by whatever route could improve aphid resistance. Second, aphids may gain more protective phages. Again, little is known about how the protective phage moves between *Hamiltonella* strains, although there is evidence for considerable variation between phages in the resistance they confer [9]–[11] and the *Hamiltonella* genome shows evidence of extensive horizontal transfer of mobile elements [23]. Third, parasitoid attack may select for aphid clones that have higher resistance leading to a change in mean population phenotype. Finally, and our focus here, improved resistance may occur within clonal lineages of aphid by changes in the abundance or genetic composition of the symbiont or phage.

The degree of symbiont-conferred resistance in pea aphids appears to be proportional to the number of *Hamiltonella* cells present. Black bean aphids (*Aphis fabae*) in their first and second instars, which contain fewer *Hamiltonella* cells, are vulnerable to parasitoids, even when they carry a protective strain of *Hamiltonella* [24]. We have also observed that individuals from one pea aphid clone (subsequently used in this study) gain little protection from their symbionts in the first and second instars (83% versus 90% parasitism; N = 116, 92) but are moderately protected from the third instar onwards (53% versus 81% parasitism; N = 98, 76). The toxin genes involved in resistance appear to be constitutively expressed rather than induced [25] and so the increase in resistance as the aphid grows may be a simple reflection of the growing number of bacteria. If there is heritable variation in cell number amongst clonal aphids then parasitism might select for greater bacterial cell densities and hence resistance. Although higher symbiont numbers have previously been associated with fitness costs [26], [27], an increase might still be beneficial if the symbionts are protective and the risk of parasitoid attack is high.

We conducted an experiment to test whether parasitoid resistance within a single clonal line of pea aphids (*Acyrthosiphon pisum*) might increase after exposure to the wasp *A. ervi* for nine successive generations. Over this timescale, we expected any change in resistance to occur because of increased densities of protective phage rather than genetic change within the aphid, bacterial symbiont, or the phage it carries. We hypothesised that the density of protective symbionts might increase through growth in bacterial numbers or through an increase in the fraction of bacteria carrying multiple copies of the protective APSE phage. Loss of phage occurs infrequently but regularly in certain aphid lines [9], [26] which suggests phage dynamics may lead to variation in copy number upon which natural selection can operate.

## Methods

### Ethics statement

The pea aphid clone used in our experiment was collected from *Lotus pedunculatus* growing on private land in Berkshire, UK in 2003 (grid reference SU 976 851), with permission from the landowner. Any future researchers wishing to use the same location should contact the owner of the land at the time of their study in order to gain similar permission to collect.

### Experimental organisms

A line of aphids was established from a single female and maintained in culture in the laboratory on broad bean (*Vicia faba*). The taxon *A. pisum* consists of a complex amalgam of host plant-associated races [28] but all are able to feed on cultivated *V. faba* [29], [30]. Diagnostic PCR was used to confirm that this clone carries the symbiont *Hamiltonella defensa*, but no other known secondary symbionts of pea aphids (see Henry *et al*. [20] for details of primers and PCR conditions used). This clone was chosen because preliminary experiments had shown it to be partially resistant to the parasitoid *A. ervi* and for resistance to increase with age. When carrying *Hamiltonella* about 50% of aphids survive parasitoid attack but this drops to less than 20% in sub-lines from which the symbiont has been removed using antibiotics (for details of antibiotic curing protocol, see McLean *et al*. [31]).

Aphids were maintained in culture and in the experiments at 20°C with a 16∶8 h light∶dark cycle, and kept in 9 cm Petri dishes containing a single leaf of *V. faba* with the petiole inserted in 2% agar gel to keep it fresh. Aphids were transferred to a fresh leaf once a week. The *A.ervi* parasitoid wasps used were taken from an inbred stock maintained in the laboratory at 20°C and a 16∶8 h light∶dark cycle for over five years, and reared on a highly susceptible pea aphid clone which lacks any described secondary endosymbionts. Female parasitoids were less than one week old when used in the experiment; all had been exposed to males and so were presumed to be mated, and had been allowed prior experience of oviposition on aphids lacking secondary endosymbionts.

### Experimental design

Twelve replicate lineages of aphids were set up, six of which were exposed to parasitoid attack for nine generations, the others acting as controls. All lineages originated from a single asexual adult aphid which had been placed on a leaf of *V. faba* in a Petri dish and allowed to reproduce for 48 hours. Twelve of the offspring were removed and used to initiate the replicate lines. Once adults, these 12 aphids were placed on *V. faba* leaves for 24 hours and their offspring kept and used in the first round of parasitoid exposure.

To initiate the exposure treatment, six adult aphids were separated and allowed to reproduce for 48 hours. Four days later, the third instar offspring were exposed to parasitoid wasps. Offspring from individual females were placed together in a Petri dish (N = 12) without any leaf material and a single female *A. ervi* introduced. The dishes were observed for up to two hours, and individual aphids removed immediately after the wasp had attacked them. We know from previous experiments in which we had exposed aphids to parasitoids and immediately dissected them that a parasitoid egg can be found in>80% of individuals.

The parasitized aphids were then kept on fresh leaves of *V. faba* for 11 days, by which time surviving aphids had begun to reproduce while those that had succumbed to the wasp's attack had become “mummies” containing parasitoid pupae. From two to eight surviving aphids from each of the six experimental lines were then removed to individual 9 cm Petri dishes with fresh leaves and allowed to reproduce for 48 hours to initiate the next generation. This procedure was repeated for eight further generations. The six control lines of aphids were maintained in exactly the same way except for exposure to parasitoids.

Up to 12 Petri dishes could be watched simultaneously and exposure to parasitoids was therefore carried out in three separate blocks for each generation, conducted sequentially on the same day. The only alteration made to the protocol during the experiment was to change the parasitoid exposure arena from a 9 cm Petri dish to a 5 cm Petri dish after the third generation. This was done to increase the likelihood that the wasp located every aphid and that every oviposition event was observed.

### Final resistance assay

After nine generations of exposure to parasitoid attack the resistance of experimental lines was compared with the controls. Over the course of the experiment, two of the experimental lines became extinct, leaving four experimental lines for the final assay.

To avoid complications involving any maternal effects all aphid lines were maintained for a tenth generation without exposure to parasitoids before the final resistance assay. Ten females were then taken from each of the four remaining experimental lines and placed in dishes to reproduce for 48 hours. Ten females were also taken from four randomly chosen control lines, giving a total of 80 dishes. When the offspring had reached the third instar, 12 aphids were taken from each dish and exposed to a single parasitoid female in a 5 cm Petri dish for two hours. All wasps were allowed access to an equal number of aphids for the same length of time. The exposed aphids were kept for 12 days and the number of parasitized mummies, surviving aphids and dead aphids recorded. The number of wasps hatching successfully from the mummies was also observed over the subsequent five days.

We compared rates of parasitism and successful wasp emergence in the two treatment groups using generalized mixed modelling techniques, implemented using packages ‘lme4’ [32] and ‘car’ [33] in R version 3.0.2 [34]. In each case, we used a binomial distribution, with an individual level error term included to account for any overdispersion in proportion data.

## Results

**Figure 1:**
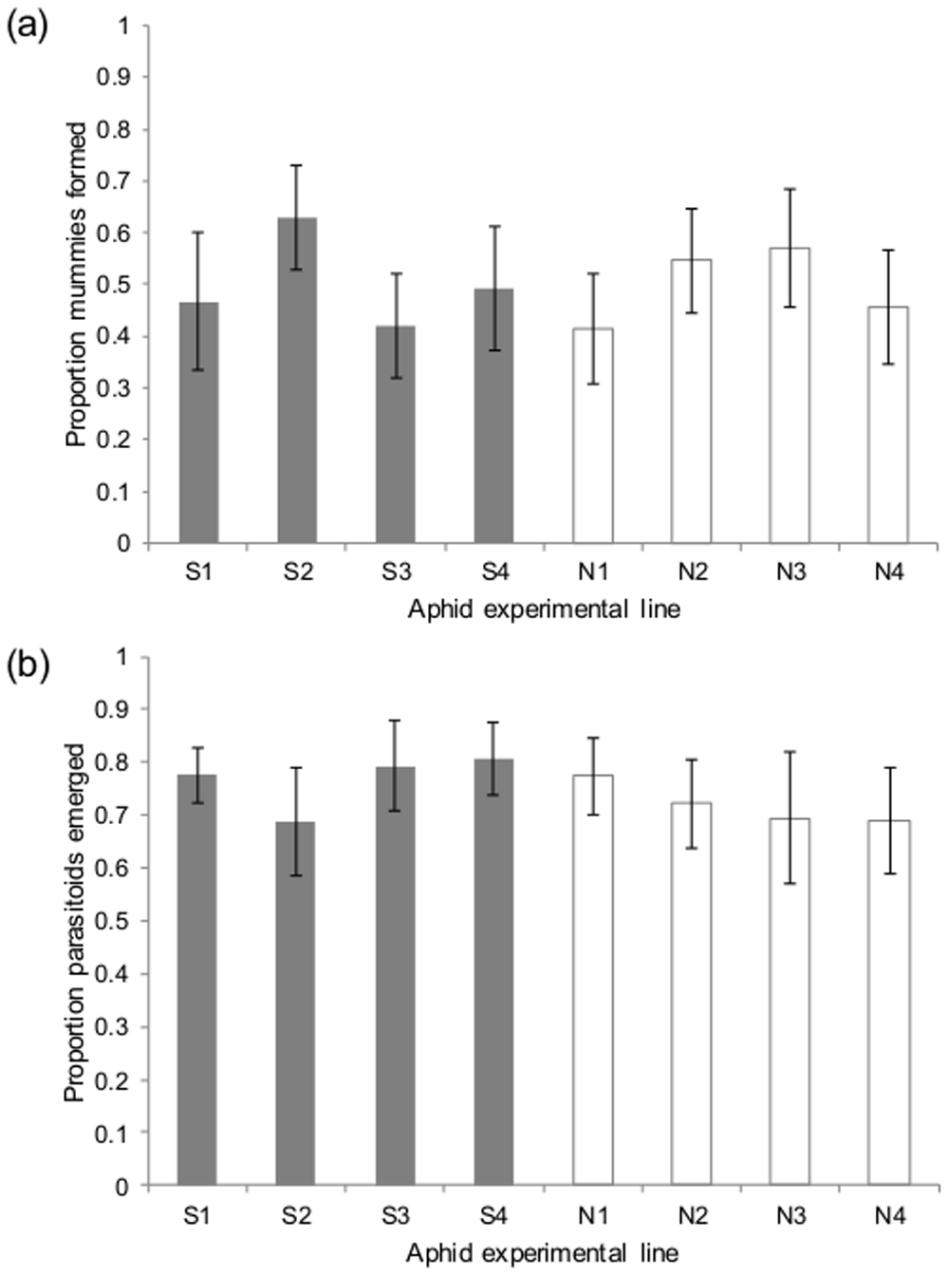
Parasitoid success rates in aphids from exposed and control treatment lines. (a) Proportion of mummies formed for the exposed treatment lines (in grey) and the control lines (in white); (b) Proportion of successful adult emergence of parasitoids from the exposed treatment lines (in grey) and the control lines (in white). No significant differences were observed between or within treatments. Error bars denote standard errors of the mean.

There was no significant difference between exposed and non-exposed treatments (χ^2^ = 0.115, d.f. = 1, P = 0.735; Fig. 1a); the average level of successful parasitism was 49.8%. This is very similar to the results of our preliminary assessment of resistance in this aphid clone before the experiment began, which found a mean of 53.0% successful parasitism. Likewise, the rate of successful emergence of parasitoids from the mummies was unaffected by treatment (χ^2^ = 0.432, d.f. = 1, P = 0.511; Fig. 1b), with emergence at 72.0% in the non-exposed group and 76.6% in the exposed lines.

## Discussion

We set out to discover whether an aphid clone that displays incomplete but significant symbiont-conferred resistance to parasitoids was able to respond to high levels of parasitism by developing increased resistance over a number of generations. We thought this most likely to occur through an increase in the numbers of the symbiont itself, or of the toxin-encoding phages which infect the symbiont and are ultimately responsible for the resistance phenotype. We find no evidence that the aphid clone was able to respond to parasitism by developing increased resistance by any mechanism: there was no difference in the level of symbiont-conferred resistance in the lines that had or had not been exposed to parasitoids.

Our results suggest that there is little scope for short-to medium-term adaptive change in resistance within a particular aphid clone without the horizontal transfer of symbionts. This conclusion must obviously be made with some caveats. First, we used only one clone of pea aphid and the single *Hamiltonella* strain with which it had been collected in the field. It is of course possible that different aphid clones would have displayed a different response, although defence against parasitoids in pea aphids seems largely [8], if not entirely [14], [35], to be driven by the properties of the symbiont strain. Given the lack of any phenotypic change in our study, we did not go on to assess using qPCR whether either bacterial symbiont or bacteriophage titre had changed over the course of the experiment. However, it is possible that a different aphid genotype or a different symbiont strain might have provided greater heritable variation in the number of bacteria per aphid, or the number of phage per bacterium, upon which selection could have acted. Second, symbiont associations may be more plastic immediately after they have come together through horizontal transfer (as observed experimentally by Russell & Moran [36]) and the natural association we chose may have been too stable to provide variation for selection. Finally, we used a single, highly inbred, line of a single parasitoid species. Symbiont-wasp genotype × genotype interactions have been observed [37] and the use of a different (perhaps less virulent) strain or species may have led to a response being observed.

Parasitoids bred on partially resistant clones of aphid have been demonstrated to evolve increased virulence [15], [38] and to respond to symbiont presence by adjusting their oviposition behaviour [16]. The absence of short-term responses in aphids is not surprising, given their asexual reproduction within a season. Pea aphids in northern Europe have only a single sexual generation per year, and even this may be dispensed with in warmer climates. As their parasitoids can have multiple generations a year, aphids would appear to be at a disadvantage in any coevolutionary interaction. However, their main defence against parasitoids aside from defensive symbionts may be escape in time and space. Aphid colonies can grow very fast because of asexual reproduction and the viviparous telescoping of generations that this allows. They may thus be able to reach a large colony size and produce many dispersive winged aphids before the majority of their natural enemies have increased to levels at which they can cause significant mortality. The type of intra-colony selection imposed in our study may thus occur rarely in the field. Interestingly, there is evidence that winged aphids are produced earlier in the presence of parasitoids [39].

The majority of work on symbiont-conferred resistance against parasitoids has, as in this study, focussed on laboratory studies. Consequently, little is known about how symbionts affect interactions between aphids and their natural enemies in the field. *Aphidius ervi* shows additive genetic variation in virulence [15], [40] and has the capacity to evolve improved performance on different aphid species in the laboratory [41]. However, there is no evidence of genetic differentiation amongst *A. ervi* that attack different pea aphid populations on different host plants, even when these host plant-associated populations differ markedly in resistance [42], [43]. If the lack of flexibility in clonal responses to parasitism that we observe is typical, then inter-clonal selection would be expected to promote resistance only on plants where this was most advantageous. The fact that pea aphid populations adapted to different host plants differ so markedly in the defensive symbionts they carry [20], [44] suggests that the selection pressure for defence must vary between host plants. Understanding the nature of this variation, and whether the symbionts influence the complex tritrophic interactions that are known to exist between aphids, plants and parasitoids, will be an interesting avenue for future research.

## Supporting Information

Table S1 Raw data used for analysis. ‘Name’ indicates the aphid line (see graphs). Where emergence is marked N/A, there were either no mummies, or an accident prevented the wasps from being assessed following emergence (two cases). Percentage parasitism was counted using the number of mummies divided by the number of live and number of mummified aphids. Dead aphids are ambiguous and so were excluded from the analysis; however, the data are included here for completeness.
(XLSX)

## References

[1] Sheldon BC, Verhulst S (1996) Ecological immunology: Costly parasite defences and trade-offs in evolutionary ecology. Trends in Ecology & Evolution 11: 317–321. 21237861 10.1016/0169-5347(96)10039-2

[2] Sandland GJ, Minchella DJ (2003) Costs of immune defense: an enigma wrapped in an environmental cloak? Trends in Parasitology 19: 571–574. 14642767 10.1016/j.pt.2003.10.006

[3] Boots M, Begon M (1993) Trade-Offs with Resistance to a Granulosis Virus in the Indian Meal Moth, Examined by a Laboratory Evolution Experiment. Functional Ecology 7: 528–534.

[4] Kraaijeveld AR, Godfray HCJ (1997) Trade-off between parasitoid resistance and larval competitive ability in *Drosophila melanogaster* . Nature 389: 278–280. 9305840 10.1038/38483

[5] Siva-Jothy MT, Tsubaki Y, Hooper RE (1998) Decreased immune response as a proximate cost of copulation and oviposition in a damselfly. Physiological Entomology 23: 274–277.

[6] Godfray HCJ (1994) Parasitoids: Behavioural and Evolutionary Ecology. Princeton, NJ: Princeton University Press.

[7] Oliver KM, Russell JA, Moran NA, Hunter MS (2003) Facultative bacterial symbionts in aphids confer resistance to parasitic wasps. Proceedings of the National Academy of Sciences of the United States of America 100: 1803–1807. 12563031 10.1073/pnas.0335320100 PMC149914

[8] Oliver KM, Moran NA, Hunter MS (2005) Variation in resistance to parasitism in aphids is due to symbionts not host genotype. Proceedings of the National Academy of Sciences of the United States of America 102: 12795–12800. 16120675 10.1073/pnas.0506131102 PMC1200300

[9] Oliver KM, Degnan PH, Hunter MS, Moran NA (2009) Bacteriophages encode factors required for protection in a symbiotic mutualism. Science 325: 992–994. 19696350 10.1126/science.1174463 PMC5473335

[10] Degnan PH, Moran NA (2008) Diverse Phage-Encoded Toxins in a Protective Insect Endosymbiont. Applied and Environmental Microbiology 74: 6782–6791. 18791000 10.1128/AEM.01285-08 PMC2576707

[11] Degnan PH, Moran NA (2008) Evolutionary genetics of a defensive facultative symbiont of insects: exchange of toxin-encoding bacteriophage. Molecular Ecology 17: 916–929. 18179430 10.1111/j.1365-294X.2007.03616.x

[12] Gerardo NM, Altincicek B, Anselme C, Atamian H, Barribeau SM, et al (2010) Immunity and other defenses in pea aphids, *Acyrthosiphon pisum* . Genome Biology 11: 16. 10.1186/gb-2010-11-2-r21 PMC2872881 20178569

[13] Laughton AM, Garcia JR, Altincicek B, Strand MR, Gerardo NM (2011) Characterisation of immune responses in the pea aphid, *Acyrthosiphon pisum* . Journal of Insect Physiology 57: 830–839. 21439291 10.1016/j.jinsphys.2011.03.015

[14] Martinez AJ, Ritter SG, Doremus MR, Russell JA, Oliver KM (2014) Aphid-encoded variability in susceptibility to a parasitoid. BMC Evolutionary Biology 14: 127. 24916045 10.1186/1471-2148-14-127 PMC4057601

[15] Dion E, Zele F, Simon JC, Outreman Y (2011) Rapid evolution of parasitoids when faced with the symbiont-mediated resistance of their hosts. Journal of Evolutionary Biology 24: 741–750. 21261770 10.1111/j.1420-9101.2010.02207.x

[16] Oliver KM, Noge K, Huang EM, Campos JM, Becerra JX, et al (2012) Parasitic wasp responses to symbiont-based defense in aphids. BMC Biology 10: 11. 22364271 10.1186/1741-7007-10-11 PMC3312838

[17] Moran NA, Dunbar HE (2006) Sexual acquisition of beneficial symbionts in aphids. Proceedings of the National Academy of Sciences of the United States of America 103: 12803–12806. 16908834 10.1073/pnas.0605772103 PMC1568928

[18] Sandström JP, Russell JA, White JP, Moran NA (2001) Independent origins and horizontal transfer of bacterial symbionts of aphids. Molecular Ecology 10: 217–228. 11251800 10.1046/j.1365-294x.2001.01189.x

[19] Russell JA, Weldon S, Smith AH, Kim KL, Hu Y, et al (2013) Uncovering symbiont-driven genetic diversity across North American pea aphids. Molecular Ecology 22: 2045–2059. 23379399 10.1111/mec.12211

[20] Henry Lee M, Peccoud J, Simon J-C, Hadfield Jarrod D, Maiden Martin JC, et al (2013) Horizontally Transmitted Symbionts and Host Colonization of Ecological Niches. Current Biology 23: 1713–1717. 23993843 10.1016/j.cub.2013.07.029 PMC3980636

[21] Russell JA, Latorre A, Sabater-Munoz B, Moya A, Moran NA (2003) Side-stepping secondary symbionts: widespread horizontal transfer across and beyond the Aphidoidea. Molecular Ecology 12: 1061–1075. 12753224 10.1046/j.1365-294x.2003.01780.x

[22] Gehrer L, Vorburger C (2012) Parasitoids as vectors of facultative bacterial endosymbionts in aphids. Biology Letters 8: 613–615. 22417790 10.1098/rsbl.2012.0144 PMC3391472

[23] Degnan PH, Yu Y, Sisneros N, Wing RA, Moran NA (2009) *Hamiltonella defensa*, genome evolution of protective bacterial endosymbiont from pathogenic ancestors. Proceedings of the National Academy of Sciences of the United States of America 106: 9063–9068. 19451630 10.1073/pnas.0900194106 PMC2690004

[24] Schmid M, Sieber R, Zimmermann YS, Vorburger C (2012) Development, specificity and sublethal effects of symbiont-conferred resistance to parasitoids in aphids. Functional Ecology 26: 207–215.

[25] Moran NA, Tran P, Gerardo NM (2005) Symbiosis and insect diversification: An ancient symbiont of sap-feeding insects from the bacterial phylum Bacteroidetes. Applied and Environmental Microbiology 71: 8802–8810. 16332876 10.1128/AEM.71.12.8802-8810.2005 PMC1317441

[26] Weldon SR, Strand MR, Oliver KM (2013) Phage loss and the breakdown of a defensive symbiosis in aphids. Proceedings of the Royal Society B: Biological Sciences 280. 10.1098/rspb.2012.2103 PMC3574403 23193123

[27] Oliver KM, Moran NA, Hunter MS (2006) Costs and benefits of a superinfection of facultative symbionts in aphids. Proceedings of the Royal Society B-Biological Sciences 273: 1273–1280. 10.1098/rspb.2005.3436 PMC1560284 16720402

[28] Peccoud J, Ollivier A, Plantegenest M, Simon JC (2009) A continuum of genetic divergence from sympatric host races to species in the pea aphid complex. Proceedings of the National Academy of Sciences of the United States of America 106: 7495–7500. 19380742 10.1073/pnas.0811117106 PMC2678636

[29] Sandström J (1996) Temporal changes in host adaptation in the pea aphid, *Acyrthosiphon pisum* . Ecological Entomology 21: 56–62.

[30] Ferrari J, Via S, Godfray HCJ (2008) Population differentiation and genetic variation in performance on eight hosts in the pea aphid complex. Evolution 62: 2508–2524. 18647340 10.1111/j.1558-5646.2008.00468.x

[31] McLean AHC, van Asch M, Ferrari J, Godfray HCJ (2011) Effects of bacterial secondary symbionts on host plant use in pea aphids. Proceedings of the Royal Society B-Biological Sciences 278: 760–766. 10.1098/rspb.2010.1654 PMC3030854 20843842

[32] Bates D, Maechler M, Bolker B, Walker S (2014) lme4: Linear mixed-effects models using Eigen and S4. R package version 1.0–6.

[33] Fox J, Weisberg S (2011) An {R} Companion to Applied Regression, Second Edition. Thousand Oaks, CA: Sage.

[34] R Development Core Team (2013) R: a language and environment for statistical computing. Vienna, Austria: R Foundation for Statistical Computing. Available: http://www.r-project.org/. Accessed 2014 Oct 26.

[35] Parker BJ, Garcia JR, Gerardo NM (2014) Genetic variation in resistance and fecundity tolerance in a natural host-pathogen interaction. Evolution 68: 2421–2429. 24689981 10.1111/evo.12418

[36] Russell JA, Moran NA (2005) Horizontal transfer of bacterial symbionts: Heritability and fitness effects in a novel aphid host. Applied and Environmental Microbiology 71: 7987–7994. 16332777 10.1128/AEM.71.12.7987-7994.2005 PMC1317397

[37] Vorburger C, Sandrock C, Gouskov A, Castaneda LE, Ferrari J (2009) Genotypic variation and the role of defensive endosymbionts in an all-parthenogenetic host-parasitoid interaction. Evolution 63: 1439–1450. 19228189 10.1111/j.1558-5646.2009.00660.x

[38] Rouchet R, Vorburger C (2012) Strong specificity in the interaction between parasitoids and symbiont-protected hosts. Journal of Evolutionary Biology 25: 2369–2375. 22998667 10.1111/j.1420-9101.2012.02608.x

[39] Weisser WW, Braendle C, Minoretti N (1999) Predator-induced morphological shift in the pea aphid. Proceedings of the Royal Society of London Series B-Biological Sciences 266: 1175–1181.

[40] Henter HJ (1995) The potential for coevolution in a host-parasitoid system. 2. Genetic variation within a population of wasps in the ability to parasitize an aphid host. Evolution 49: 439–445. 28565084 10.1111/j.1558-5646.1995.tb02276.x

[41] Henry LM, Roitberg BD, Gillespie DR (2008) Host-range evolution in *Aphidius* parasitoids: Fidelity, virulence and fitness trade-offs on an ancestral host. Evolution 62: 689–699. 18182071 10.1111/j.1558-5646.2007.00316.x

[42] Hufbauer RA (2001) Pea aphid-parasitoid interactions: Have parasitoids adapted to differential resistance? Ecology 82: 717–725.

[43] Bilodeau E, Simon JC, Guay JF, Turgeon J, Cloutier C (2013) Does variation in host plant association and symbiont infection of pea aphid populations induce genetic and behaviour differentiation of its main parasitoid, *Aphidius ervi*? Evolutionary Ecology 27: 165–184.

[44] Ferrari J, West JA, Via S, Godfray HCJ (2012) Population genetic structure and secondary symbionts in host-associated populations of the pea aphid complex. Evolution 66: 375–390. 22276535 10.1111/j.1558-5646.2011.01436.x

